# The New York Institute of Stomatology

**Published:** 1896-02

**Authors:** 

**Affiliations:** The New York Institute of Stomatology


### THE NEW YORK INSTITUTE OF STOMATOLOGY.

A general meeting of the Institute was held on the evening of November 26, 1895, at the residence of Dr. George S. Allan, No. 51 West 37th Street, the President, Dr. Benjamin Lord, presiding.

The minutes of the last general meeting were read.

*The President.*—The notice of this meeting calls for communications on theory and practice, as our first business in order. If any of the gentlemen have anything to communicate we will be very happy to listen to them.

*Dr. W. St. George Elliott.*—Allow me to call your attention to a new operating-table heater that I have recently designed and made. The object aimed at is to heat the syringe-water, keep syringe itself warm, heat hot-air syringe, soften gutta-percha, and keep mouth-mirrors warm, so that moisture will not condense upon them.

The apparatus as you see is very simple, consisting of a copper plate two by six inches, on one side of which there is a Bunsen burner. Over this burner is a copper disk to catch the heat, which is then conveyed by the copper support to the plate below, and thence to the glass of water, and up the copper pedestal which supports the syringe. The mirrors rest on the copper plate. The gutta-percha is softened on a German silver disk, placed for a moment on the heated copper disk. The warm-air syringe is heated by being placed in the copper tube support to the heating disk. The flame of the Bunsen burner never jumps down, and the blue flame only requires to be one-quarter inch in height. I have used both alcohol and kerosene for years, but they are liable to give out or burn out at a critical time. I see no objection to gas.

*The President.*—Are there any other communications on theory or practice? If not, I will say that I have some instruments here that have been spoken of occasionally during the past year, for removing tartar from the teeth, and which are thought to be an improvement on anything before used. They are simply the ordinary sickle instruments with the edges grooved. It recently occurred to me that the edges could be sharpened better by a very fine serrating file than with a file that leaves a smooth surface. I will pass the instruments around for examination. They are all made on the same principle, only of different thicknesses. In the very thin instruments the grooves should be made before the instrument is reduced to the desired thickness. The grooves, as may be seen, are in both edges, so that the pushing or pulling effort are equally effective. It is found that these instruments are excellent for finishing the margins of fillings, as well as for the removal of tartar, for which they are especially designed. The edge is such that it is sure to take hold of anything that we may wish to remove. Probably we have not had an instrument before with which we could be sure to discover and remove any foreign substance from the necks and roots of the teeth. I now take pleasure in introducing the essayist of the evening, Dr. Henry W. Gillett, of Newport, Rhode Island.

*Dr. Gillett.*—Mr. President and gentlemen, years ago, it seems to me, when I was a student and was accustomed to read the reports of the society meetings and doings of the profession in New York, I was greatly interested, and felt that they had reached the acme of the profession; and to-night I feel it to be a great honor to have reached a point myself where I have the privilege of addressing this Institute and of trying to say something that may be interesting, even if it be not entirely new. And I wish to say that, finding I had rather too much material to put into a paper to be given in one evening, I have taken the liberty of modifying the title as printed in the programme by adding the word living; making the title Electrical Osmosis in the Treatment of Living Dentine. In doing that I have simply thrown out, from lack of time to consider it, the process of bleaching dentine and of bleaching teeth generally, by the aid of the electrical current and solutions of bleaching agents.

(For Dr. Gillett's paper, see page 65.)

*Dr. Gillett.*—This instrument is manufactured by the Electro-Therapeutic Company, 32 East Twenty-third Street. I have here a graphite rod with wires attached, so that it may be put in the working circuit in place of the patient. By doing this I can show the method of turning in the current. By watching the milliampère metre-needle the regularity with which the current is added can be understood.

*The President.*—Gentlemen, this very interesting paper upon a most important subject is now before you for discussion.

DISCUSSION.

*Dr. Geo. S. Allan.*—Before we proceed to the general discussion of Dr. Gillett's paper, I hope the president will call upon Dr. Morton, who has given this subject a great deal of attention from a medical stand-point, and has succeeded in anæsthetizing the soft tissues, while, as far as I can understand, Dr. Gillett has not been successful in that direction. As dentists it is important for us to know how we can apply this treatment to the soft tissues with the same success that Dr. Gillett has achieved in treating the dentine.

*The President.*—We are very highly favored, indeed, in having Professor Morton with us this evening. Gentlemen, I present Professor W. J. Morton, M.D., of the Post-Graduate Medical School and Hospital of this city.

*Dr. Morton.*—I assure you that it gives me much pleasure to be with you to-night; the more so as I have had the privilege of listening to an admirable and well-presented paper. Dr. Gillett certainly has left very little for me to say, assuredly so in certain directions, even if I were to attempt to add to what he has said. There were several points in the paper that attracted my attention as he went along and that I tried to save in my mind for reference. One of the first thoughts that occurred to me was the extreme pleasure which it gave me to see this admirable and beautiful principle of electricity and medicine and its combinations brought into a practical use. I have long been familiar with these properties of the electric current. As long ago as 1879 I began to work on this subject of cataphoresis and had electrodes made, and I think that to-night we have had laid before us principles and practice which will not die in a lifetime. I recognize in it one culmination of an enormous amount of work in the way of experimentation that has been done, and also in practical operations which have not come out sufficiently in public. It remains for the dental profession to establish one practical use. And it is a curious fact that to the dental profession really belongs the credit of procedures, both general and local, in their initiation, which have tended to annihilate pain, whether it is done with nitrous oxide gas, or with ether, or with chloroform, which was merely a later substitute for ether, and therefore marked no new era. All of these processes have had a practical beginning with dentists, and therefore, because of the starting of this method in this way, more attention may be attracted to it in medicine. Years ago I tried to produce cataphoric anæsthesia with chloroform, following the experiments of Adamkiewicz, of Vienna. I read also the experiments of Dr. B. W. Richardson and tried to repeat them, but have never been able to do so in a practical manner. I do not, therefore, think it is possible to make a reliable and practical cutaneous cataphoric anæsthesia with chloroform or ether. In 1890 I was much attracted by the experiments of Edison in introducing lithia salts in cases of gout by cataphoric action, for the purpose of removing gouty accretions in the joints, in which he had some success. From that time quite a number of experimenters took up cataphoric medication, but it has never been much used in general practice, either in medicine or dentistry. Still it has been effectually used in cases in private practice since 1890. In 1890, Dr. Peterson took up the subject, and you have heard in the paper what he did. He tried different alkaloids, aconitia, hydrochlorate, or muriate of cocaine, etc., with the success that has been described. The wonder to me is that in all this time cataphoresis has received so little attention, and that there has not been until recently any signs of progress made in cataphoric anæsthesia, or in general introduction of medicines into the system.

With regard to the point raised by Dr. Allan, as to the effect upon the soft tissues, I will say that my experience has been mainly upon those tissues. I have taken an electrode of one inch in diameter, and at other times a three-inch electrode, and, having placed it upon the skin, the negative pole placed at some indifferent point, have made the skin perfectly numb, turning it pale, and giving it a shrunken appearance, this and the anæsthesia enduring for fifteen to thirty minutes. No harm is done to the skin or the mucous membrane by the introduction of cocaine in that way. There are many difficulties in making these practical experiments upon the soft tissues which are not met with in the case of sensitive dentine. I think some of the failures in introducing medicine through the skin or the mucous membrane have been due to a non-recognition of the fact that in cataphoric conduction the surface to be affected must be very close to the metal. The intervening space between the metallic plate and the skin has usually been filled with sponge; and the greater the distance thus intervening the less the cataphoric effect; the less the distance the greater the cataphoric effect. In order to get cataphoric anæsthesia of the skin, the metal should be placed almost upon the skin, with some thin substance holding the medicine between, such as a piece of blotting-paper, and then the cataphoric effect is quite pronounced. I think some of the failures that have been made in the past have been due to overlooking this fact. I saw to-day a couple of rubber cups where the metal conductor was about half an inch from the tissue. Certainly there would be little cataphoric effect in a case like that. In the application to dentine the platinum wire electrode, as good fortune would have it, comes unavoidably close to the tissue to be affected. A question that arises is, Does the effect upon soft tissues extend deeply? I am certain that it does. But I do not think there would be much chance of anæsthetizing the pulp or the locality of the apex of a tooth in such a way. To effect this latter when a cavity exists, I would make application from the cavity, and the drug is carried cataphorically through the pulp cavity to the end of the tooth and to the tissue there situated. By this same pathway, also, other medicines—say chloride of zinc, for example—may be carried to the end of the roots of the tooth and out through them to surrounding tissue, and thus afford a valuable means of introducing local remedies to diseased parts otherwise difficult to reach. There is a case reported by Dr. Peterson where the fifth nerve was affected and in which the relief from pain was continued for ten or twelve hours. This might be in favor of there being a deep effect, or it might be explained in another way.

An electrode to produce cocaine anæsthesia electrically for the extraction of teeth is familiar to many. A positive pole is placed upon the gums upon one side of the tooth and a negative pole upon the gums upon the other side. By no possibility could this electrode anæsthetize more than the gums on one side,—namely, that on the positive side,—and hence a painless tooth extraction would be by means of this electrode out of the question. The electrode must present a positive pole to *both* sides of the tooth. There is a certainty of getting a deep cocaine effect upon the gums in this way.

Iodide of potassium can be introduced through the skin, placing it upon a piece of blotting paper between the skin and the electrode, and in twenty or thirty minutes detect the iodine in the urine. To show how I have employed this method in medicine, I will say that within the last year I have treated some cases that required the use of mercury, introducing it in this way instead of by the inunction method. Recently a woman came to us with suspicious indications of syphilis. She was referred to me at my electro-therapeutic clinic, and I was at first quite at a loss what to do, for in this clinic I use only electricity as an agency for cure. It occurred to me that I would treat the case by the introduction of mercury by means of electricity. I did so, using two foot-tubs, with corrosive sublimate in one, making it the positive pole, and salt and water in the other, making it the negative pole, and turning on thirty to forty milliampères. On the sixth day the patient was salivated, and we had all the evidences that the medicine had produced a constitutional effect.

I have nothing in particular to say about this apparatus presented that is called an "adapter." The word adapter I do not like to criticise, but I think our old friend rheostat has become familiar to us, and I like to hear things called by their right names. I have not seen the inside of that machine, and there may be some reason of which I am not aware for calling it an adapter, but it certainly is a most admirable rheostat. I spent an hour investigating it this afternoon. It is an arrangement that cuts down the flow of the current like a stop-cock in a water-pipe, and enables one to use, practically, the equivalent of one cell or four or twenty, as he likes, and to use very small fractions of current. The advantage of this is, that where cells having a voltage, say, for example, of two volts each, are used, a jump has to be made of two volts at a time; but with this rheostat the current is regulated so as to be perfectly smooth. Another thing: In talking of the strength of currents, the essayist uses the word volts to express the terms of measurement of the gradation of current employed in making successive applications. Generally, when talking of currents, ampères, or fractions of ampères are the terms employed. One can talk about volts if he pleases, and it may in one sense be fairly correct, but the terms ampère and milliampère are more correct. It is equivalent, in using the word volt, to say, I use one, or five, or ten cells, etc.; and that is not the real question. The question is, How much strength of current is used,—that is, how many ampères or fractions of an ampère.

I think, gentlemen, that is all I have to say at present. If, later on, I shall be able to give any information on the subject, I shall be glad to do so. Dr. Gillett has collected a great deal of information on this question, and it would be almost superfluous, certainly as to its application in dentistry, for me to try to add to it. Not that electrical anæsthesia or "cataphoresis" is new. We all know that tissues can be actually benumbed by means of an electro-cocaine application; but Dr. Gillett has demonstrated that sensitive dentine can be benumbed, and that is a very happy thing to do. I think the credit is due to the people who do the thing, not to those who merely theorize, and that one honest demonstration is worth about a ton of theory, and such demonstrations we have had, in spite of the scepticism of a great many people. The facts that Dr. Gillett has brought forward to-night will live a long time.

*Dr. Howe.*—I have been very much interested in Dr. Gillett's presentation of this subject, because I have made some similar experiments, and I am glad to endorse what he has said. But I think that Dr. Gillett has not given due credit to Dr. Westlake, who published in 1892 the results of experiments which he had made, and in which he accomplished the same results. His paper was read before the Dental Society of the State of New York, and was published in 1892. I experimented in this line a little more than a year ago. I obtained some information from Dr. Peterson, who has been so largely quoted from, and began experiments to produce anæsthesia of the tooth-structure. In two or three of the first cases the cavities were rather inaccessible to electrodes, and I did exactly what Dr. Gillett found to be rather impracticable. I produced anæsthesia of the dentine by applying the electrodes to the gum tissue on each side of the tooth-roots, as nearly opposite the apices of the roots as possible. A solution of cocaine was placed on the sponge in one of these small soft-rubber cups and a salt-water solution in the other. The first application that I made was successful in twelve minutes in producing complete anæsthesia of the dentine in an extremely sensitive cavity. This is the electrode that I used in that and several other cases since. You will perceive that it is home-made and rather crude. I never knew of any irritation of the mucous membrane, or any discomfort following such applications. In my later applications of the electric current for its cataphoric effects, I have applied the anode to cotton, with a solution of cocaine in the cavity, and have generally put the cathode in the patient's hands. This method has taken, I think, rather more time than the former one of sending a short circuit through from one side of the gum to the other across the roots of the tooth. I will mention in connection with this subject that this experimentation suggested to me the possibility of causing increased circulation and nutrition in the gums by applications of the faradic current. In experiments of this kind I have used my finger for the negative electrode, applying it to the gums, and have placed the positive in the patient's hand. My finger has been insulated by covering it with a soft-rubber finger-stall, having a hole cut in it near the end, corresponding in location to the palmar surface. These applications of the faradic current have been followed by a very satisfactory stimulation of nutrition, in two or three cases, in which the patients were willing to follow up the treatment. But in resuming the special subject, I would say that Dr. Gillett's experience, that the consumption of time in anæsthetizing dentine by cataphoresis was not so great as to make him feel that he was not compensated, has not exactly coincided with mine. I must confess that I did feel after a while that I was getting into deep water, because I was consuming so much time, and not accomplishing enough to pay for the loss thus incurred. This would not be so, except for the demands of patients that their work be done painlessly, whether in the operator's judgment the severity of the operation requires anæsthesia or not. The question of time has caused me to use cataphoresis very little of late. To-day, however, I happened to have a patient in the chair who had a cavity that was so sensitive that I could do nothing with it, and I applied the current for about twelve minutes, with a solution of cocaine in the cavity, just as Dr. Gillett has described, and it reduced the sensitiveness very much indeed; not completely; but, if I had persisted longer, the anæsthesia would have been complete; there is no question, gentlemen, about that. There has been suggested to my mind a doubt as to the desirability of using the Edison current for these operations, notwithstanding the interposition of a rheostat. There is a possibility of a two-thousand-volt wire touching the wires of the system that we are connected with; perhaps it might occur in a burning building, or in some unexpected displacement by other means; and such possibilities ought not to be ignored. There would seem to be no need of using the street current, for I am informed by Dr. Peterson, with whom I have talked within a day or two, that the chloride of silver dry-cell battery is entirely satisfactory and can be used for several years without renewal.

*Dr. C. A. Woodward.*—I called at Dr. Gillett's office in Newport at a time when he had been applying this current for some minutes to a patient's tooth, and after speaking with me he returned to the patient and began excavating the cavity. The patient winced, and said she was suffering some pain, but not as much as she had before the application. Dr. Gillett repeated the application, and in the course of about five minutes she declared there was no feeling whatever from the cutting, which was proceeded with very vigorously.

*Dr. M. L. Rhein.*—I made a special visit to Newport to see Dr. Gillett, and I saw him use this method on a patient very successfully, although I don't suppose it was a very good case, because it was a woman somewhat advanced in life, with the class of teeth that are not as a rule very sensitive; but to a careful observer there was absolutely no question of the complete anæsthesia of the cavity on which he was working. He not only demonstrated it to me in that way, but he made an actual demonstration of the method on one of my own teeth. Unfortunately for the object in view, I had no cavity in any tooth in which to demonstrate the method, but I had a well marked erosion on a superior central incisor that at certain times becomes more or less sensitive. The rubber dam was adjusted over this tooth, and I could distinctly feel the application of the electricity in connection with the solution of cocaine that was used. It was, however, not painful, and at the expiration of the time allotted by Dr. Gillett there was absolutely no sensation on the surface of the tooth, though before he made the application, I felt very unpleasantly the passing of a burnisher over its surface. One of the points which he brought up in the paper might be of interest in connection with my observation of the condition of my tooth after I left his office. For two days it had that well-known numb-like feeling. This experience of mine is very much at variance with that of others as to the speedy loss of the anæsthetic effect. My own view of the matter is that the duration of the anæsthetic effect is more or less dependent on the density of the tooth to which it is applied. After this peculiar feeling had passed away, a condition of hyperæsthesia ensued, which lasted fully forty-eight hours, and for that length of time it was extremely uncomfortable in the mouth. I happened to meet Professor Morton in connection with some little experiments in a similar direction, and mentioning this to him he corroborated my own observation of the fact that we are very apt to get a condition of hyperæsthesia following the condition of anæsthesia. It is an interesting point for us to consider, because we are apt to meet with it in the use of this method, and I myself would be pleased to hear from Dr. Gillett what his experiences have been with regard to any subsequent hyperæsthesia. The subject of local anæsthesia is one that has interested me personally for a number of years, and I have had considerable success in certain forms of cavities. Some of the gentlemen present may recollect an article of mine on the use of intense cold for that purpose, a temperature of seventy degrees below zero applied for five seconds by means of the chloride of methyl. In my experience in the use of intense cold in a large number of cases there ensued this condition of hyperæsthesia that I have spoken of. I think the experiences of Dr. Gillett on this point would be very instructive.

*Dr. Gillett.*—With regard to the point that Dr. Rhein raises, I have had many cases where the application was much more prolonged, and with deeper effects obtained, without noting a numbness of the teeth for more than a few hours. In my own tooth I did not note anything of that kind. I do not remember whether my mind was acutely attentive to that point or not. Nor did I have any hyperæsthesia afterwards. I have been using this method regularly in my practice since March, and I have not seen any case where there was hyperæsthesia that I was able to distinguish from the ordinary hyperæsthesia that might come after filling a sensitive tooth.

*Dr. Rhein.*—I want to say that it was not in my experience a bad condition at all, or one that could have exerted a serious effect on the tooth; it was merely an uncomfortable sensation, it might even have been called an unpleasant feeling, by which my attention was constantly attracted to that particular tooth. But the interesting point of that is that Dr. Gillett may have attributed to the operation on the tooth what was merely the after-effect of the anæsthesia. Perhaps Dr. Howe or Professor Morton may be able to give us some information obtained in the course of their observations on that point.

*Dr. Morton.*—In local anæsthesia there is always a reaction to a more sensitive condition, or hyperæsthesia, which will last about an hour.

*Dr. Allan.*—Has Dr. Morton employed this cataphoric method in the mouth at all?

*Dr. Morton.*—Not on the dentine of the teeth.

*Dr. Allan.*—On the soft tissues?

*Dr. Morton.*—Yes.

*Dr. Allan.*—Using an apparatus similar to Dr. Gillett's?

*Dr. Morton.*—I use a common rheostat.

*Dr. Allan.*—May I ask if the whole system of procedure was similar to his?

*Dr. Morton.*—Identical. It would not vary so far as I have understood him and without further knowledge of his technique.

*Dr. Allan.*—How does Dr. Morton account for the failure that Dr. Gillett has had in applying cocaine through the gum?

*Dr. Morton.*—I don't understand that. Dr. Gillett says he did make the gums numb. What I understood him to say was that he did not reach the pulp cavities of the teeth, which are a long way from the gum. The effect did not go deep enough; and I should say he never could get cocaine to go deep enough through the gums to affect the pulp of a tooth at the root, because I think it is too far away and the circulation is too active. One of my own experiments in 1890, when I made some contributions to the subject, was to place a ring around the part I wished to anæthetize, and with pressure upon it cut off the circulation of the skin so that there was no current of blood flowing in that part. In that way the cocaine went into the tissue and was not washed off by the general circulation, and the effect produced was much greater than otherwise. That effect has been repeated, and that process is in vogue at present to a certain extent. In the case of the pulp in the root of a tooth, it is quite distant from the point of application, and what little cocaine gets there may be washed along and away in the blood-stream. But I do not understand that the gums cannot be made numb. If the electrode is properly constructed they certainly can be. I have made both the skin and the gums absolutely pale and anæsthetic with cocaine. But, better than by rings and other means of circumscribing circulation by pressure, chemical agencies may be used to restrict the action of cocaine to local areas of territory.

*Dr. Wendell C. Phillips.*—I regret very much that another engagement kept me away from this meeting until Dr. Gillett had finished the reading of his paper, but I have heard enough of the discussion, and I am familiar enough with the work that Dr. Gillett has been doing to lead me to surmise the ground that he has covered to-night. I became exceedingly interested in the subject of cataphoresis in the obtunding of sensitive dentine, partly from the fact that I am interested in the general subject of electricity apart from this particular application of it, but mainly that it was during a visit at my office where I was explaining some experiments I had been making with cataphoresis in my throat and ear work, that Dr. Gillett suggested that it might be used in obtunding sensitive dentine, and the first experiment followed, and I have watched his work with a great deal of interest from that time up to the present. I remember that the first experiment, which was made under rather unfavorable circumstances with the Edison controller, which is not adapted to this work upon the teeth, was so successful that I said to Dr. Gillett that cocaine cataphoresis is destined to revolutionize dentistry ; and I believe that prophecy is about to be realized. I must say, in answer to one or two questions that have been raised to-night, that my judgment is, if electricity is to be used at all, the sooner we master the working of the street current and use it, the better.

Use the current that is always present, and that has a certain known voltage, and master the necessary information to be able to handle that current. It is the most convenient and it is always under control if one has a proper controller with which to use it. After some years experience and work in this line I have thrown out, with one exception, every electrical apparatus not used with the street current; that exception is the electric cautery storage battery. It is but fair to say that in recent years there has been great improvement in these various appliances, but the street current gives most satisfaction, but one must use the other forms when the street current cannot be obtained. With regard to the question raised by Dr. Howe in connection with the use of the Edison current and the possibility of a high tension current coming in contact with the wire in use, I don't think any intelligent electrician who has made a study of this subject, would manufacture an adapter without providing for this contingency. It has been overcome in the controller seen here to-night, for it has a little device that would immediately burn out and shut off the current.

Now as to the question of cocaine and its action: I have only had experience in the use of cocaine in my practice in the treatment of diseases of the nose, throat, and ear. We use great care to prevent the physiological effects of the drug, because there are certain people who are peculiarly sensitive to its action. It not infrequently happens, that a very small amount of a very weak solution will throw a patient into a state bordering upon collapse. We always strive to localize its application as much as possible, and not allow it to get into the post-pharynx more than possibly can be avoided, and one should always be supplied with cardiac stimulants. Patients, however, recover in an hour or so. Probably the best way to use cocaine in dentistry is to introduce it on a pledget of cotton in the cavity of the tooth, not allowing it to come in contact with the gums, for if it does, physiological effects in occasional cases may follow and cause some alarm. It stands to reason that it will be almost impossible to force enough of the cocaine into the general circulation through the dentine of the tooth to produce any physiological effect whatever. I don't know whether the method that Dr. Howe used is better than Dr. Gillett's, but my opinion is that Dr. Gillett's method is the better one, because it sends the cocaine through the dentine in the cavity, and it goes where it is wanted. It also has the other advantage that it will not be apt to produce any physiological effect, and that would seem to be very much in favor of Dr. Gillett's method. If Dr. Howe will allow me to make a criticism of his electrode, it is that he uses a soft rubber mouth-piece, and I doubt very much whether it can be used in the mouth without objection in the matter of cleanliness. I should suppose that the method used by Dr. Gillett would be much cleaner in its application, for the negative electrode is outside the mouth, and the positive electrode can be made of some smooth metal, such as platinum. Platinum can be rendered absolutely aseptic before it is placed in a patient's mouth again. Some of us have learned a lesson by experience, and I am led more and more to feel the necessity of having hands, instruments, apparatus, cuspidors, etc., strictly aseptic. People are getting educated upon this question of cleanliness to such an extent that they demand that we have every instrument aseptic; and I don't blame them very much, because there are dangers that we little realize of carrying disease to these delicate tissues.

Now, as to the use of this particular apparatus known as the fractional volt selecter, and manufactured by the Electro-Therapeutic Company, I look forward to its use in my practice, because of the exact control it gives of the voltage and current. In that instrument we have one by which we can turn on a fraction of a volt of current, and know exactly what we are using. Since I began to make some experiments in cataphoresis, I have up to this time used three or four different electrodes. One is a zinc electrode, another a copper electrode. I have used solutions of copper and zinc, and I cannot say that I have noted any effects from them. I have used most effectually the pyrozone in ether solution twenty-five per cent., to destroy germs in the crypts of the tonsils, and in the posterior and superior pharynx. I have been told, and it has been demonstrated two or three times in my presence, that with this volt-selecter the current can be applied painlessly, and without stopping to lower the voltage the electrode can be removed without giving pain. In this principle of the apparatus, it seems to me, lies its greatest usefulness, for it must be remembered that, as ordinarily used, the galvanic current causes pain when applied to a tooth, and it would be folly to attempt to relieve one painful condition by substituting another.

*Dr. Howe.*—I think Dr. Phillips misunderstood somewhat my use of that electrode. It was purely experimental, and is not now in use. The experiments were carried on during about a month. The electrode is made so that the rubber cups come off, and the sponges are easily renewed, after each use, but if I had continued to use it in practice, it would, of course, have been necessary to have it made so as to be more readily cleansed. I would like to say, Mr. President, that I infer from something that has been said, that perhaps it was not understood that anæsthesia of the dentine was produced in my experiments, in as short or a shorter time by passing the current across the roots of the tooth, through the gum, as it was when the positive electrode was applied in the cavity. Of course, there may be reasons why that is not the best way, but it is a practicable method, and I think would sometimes be found to be the easiest way of accomplishing the desired result.

*Dr. Phillips.*—There is one question I wish to speak about if I may be allowed, and that is the length of time that cocaine has any effect upon the tissues. I think Dr. Morton partially covered that ground. The effect of cocaine upon the tissues and membranes of the nose is to contract the arterioles. The anæsthesia lasts a varying time in different cases, according to the amount used, and partly depending upon the patient himself, his resistance to the effect. After a time there is a reaction resulting in a decided relaxation of the arterioles and hyperæmia of the tissue. This effect lasts an hour or two only. The effect of cocaine upon the tissues would be entirely gone within a period of from one to three hours. Future experiments may demonstrate the length of time which cocaine anæsthesia will effect the dentine.

*Dr. Allan.*—Dr. Phillips is quite right in regard to the use of the electric current from the street wires. It is something definite and certain. In the use of the current for dental purposes I have a rheostat that I have used a few weeks, which cuts the current down for use in the mouth-lamp perfectly. The Edison Company have promised me one that will work the cautery as soon as it is completed. When they have succeeded in getting a finished instrument we will banish the battery. I have a storage battery, and it has been an unmitigated nuisance ever since I have had it. I don't quite understand why we do not have electrolysis as well as cataphoresis in the use of electricity and cocaine upon the tissues of the mouth. Electrolysis is a very different action from cataphoresis.

Electrolysis in the use of cocaine, as Dr. Gillett proposes, would mean the decomposition of the cocaine, not the forcing of it directly into the substance of the tooth. I don't quite understand that. But to us dentists this paper of Dr. Gillett's has another point of interest. It has been a moot question with us as to what was the exact nature of the contents of the dentinal tubules. No histological preparations have demonstrated the exact nature of the contents of the tubuli. Now, if this series of experiments proves anything, it does prove that there must be nerve-filaments in the dentinal tubules, otherwise it is impossible to account for this action of the cocaine.

*Dr. Jackson.*—I have been searching in my mind for some reason for the loss of the soft tissue that Dr. Gillett has spoken of, when the electric current is applied with cocaine to the gum. I would like to ask if most of the cocaine solutions in the market do not have an acid reaction, and, if the solution is acid, would not that account for the sloughing of the gum by its introduction into the tissues? Does Dr. Morton think that could be so?

*Dr. Morton.*—Just what the action of the solution would be, I really do not know, but it would scarcely cause sloughing of the gum. It was probably the strength of the current that did it, for if that is very strong, an electrolytic effect is produced. We have to avoid this over-powerful electrolytic effect, and use only sufficient current strength to produce penetration of the medicine without causing any injury to the tissue. This can easily be done.

*Dr. Jackson.*—I was trying to determine whether the presence of the acid would not account for the loss of gum-tissue. I have tested several solutions, and invariably found acid present.

*Dr. Gillett.*—Dr. Phillips says that a cocaine solution should be neutral. I have tested several solutions and found them slightly acid.

*Dr. William J. Evans.*—With regard to the acidity of cocaine solutions, I may say that very frequently cocaine solutions are prepared with salicylic or boric acid, for the purpose of keeping the solutions aseptic. The solution of anhydrous crystals of hydrochlorate of cocaine is neutral. It ought to be taken into account, however, that acid collects, out of decomposed tissue, at the positive pole, and alkalies at the negative pole during the application of a galvanic current.

I would like to ask Dr. Gillett a question in connection with this volt-selecter. As I understand electricity, the first knowledge of it is in connection with volts, the next in connection with resistance, or ohms, the next with the measurement of the current in ampères or milliampères, and the next in the measurement of the quantity of the current passing in a definite length of time. Now, I understand that electricity is very nearly related to heat, and we have a unique device here, in this fractional volt-selecter, which presents a rather amusing feature to me. We have something which cuts one hundred and ten volts down to one volt, and apparently there is something done with the electricity, which, if it is heat, is not hot. I would like a little explanation on that point.

*Dr. Gillett.*—I think, gentlemen, that the question carries me rather deeper into practical electricity than I can safely venture. Very likely Dr. Morton will be able to suggest a better answer than I can. I will say that in the practical working of this apparatus it does not go beyond the point of being fairly warm. In five, or perhaps ten, minutes, it becomes as warm as it would be in half an hour or an hour. If I understand rightly the mechanical details of its construction, it is made in this size that it may not collect heat; otherwise it could be made considerably smaller. These holes near the ends of the cylinder are put there for the purpose of allowing the heat to diffuse itself in the surrounding air, and with the aid of that diffusion of heat it does not become sufficiently hot to be objectionable in practical use.

There were several points raised in the discussion upon which I might say a word. First, I wish to express my appreciation of the very kind treatment which has been accorded me this evening. Regarding one point that Dr. Morton brought out, I would say that it would seem that one of the reasons for success in the application of this process to the dentine is also a reason why we have not succeeded in getting ordinary absorption or osmosis in dentine. The absence of active circulation in the dentine enables us to localize and intensify the effect.

The rubber cups that Dr. Morton speaks of are very nearly like those that Dr. Howe has shown. Dr. Morton took some exception to my use of the word volt or voltage, when describing the current. It is possible that Dr. Morton fails to appreciate the fact that in the use of the current through dentine it is almost impossible with the Kennelly milliampère meter to ascertain definitely what current we are using, because it is so small. A very large percentage of successful cases have been cases where the quantity of the current registered on a milliampère meter like this one has been below one milli, and in many successful cases there has been absolutely no current recorded on the milliampère meter.

*Dr. Morton.*—Then it is a bad instrument.

*Dr. Gillett.*—The fact is that a record of that quantity cannot be obtained.

*Dr. Morton.*—Then get another instrument. The reason Dr. Gillett gives shows it to be a very poor one.

*Dr. Gillett.*—I am glad to learn that there is a better milliampère meter. I have felt the need of it. Not having it, and the volt index of the adapter being conveniently at hand, I have fallen back upon an approximate statement of voltage as the best means of making a comparison. With regard to what Dr. Howe said, I have no desire to take away any credit that is due to others. My remarks concerning Dr. Westlake should be taken only as my personal estimate from my understanding of the matter. I may be entirely wrong in my estimate of what he has done. At present I think I am right. In regard to the matter of time consumed, I find in many cases that I save time, because I can work so much more rapidly after I have the sensitiveness of the tooth under control. Oftentimes the assistant can as well get the tooth ready to work upon as the operator himself. As to the use of batteries, it seems to me only a question of personal choice. I get satisfaction from the use of the Edison current, and I believe this apparatus protects me and my patient. I have found that this apparatus is very much less painful than one in which the rheostat is placed in different relations. The case quoted of the boy who went to sleep under it is a good illustration of the painlessness of the process. With regard to the question of acid reaction that was raised, one reason why I selected chloride of sodium for my experiments was on the assumption that, if any of the anæsthetizing effect was due to an acid condition at the positive pole, I would get about the same conditions from chloride of sodium and the muriate of cocaine. With the sodium chloride solutions I got no results, except irritation and pain. If these results were obtained by electrolysis and the formation of hydrochloric acid, as suggested, we ought to have the same results from the electrolysis of chloride of sodium, as there would be free chlorine and free hydrogen from the splitting up of the salt and the water, and consequently hydrochloric acid as a product. As a matter of fact, the current used is so small in quantity that the electrolysis is reduced to a minimum and need not be considered.

*Dr. Woodward.*—Mr. President, I move a vote of thanks of this Institute to Dr. Gillett for the exceedingly able and interesting paper that he has given us to-night; also to Professor Morton and Dr. Phillips for their kindness in coming out this very stormy evening to assist us in discussing this question.

Dr. Woodward's motion was carried unanimously.

Adjourned.

S. E. Davenport, D.D.S., M.D.S.,
*Editor The New York Institute of Stomatology.*

